# Cohort definition concerns in a TriNetX study comparing adolescent and young adult sleeve gastrectomy outcomes

**DOI:** 10.1016/j.obpill.2026.100349

**Published:** 2026-10-30

**Authors:** Kian-Hwee Chong, Joshua Wang

**Affiliations:** a Department of Surgery, Taipei Tzu Chi Hospital, Buddhist Tzu Chi Medical Foundation, No. 289, Jianguo Rd, Fuxing Village, Xindian District, New Taipei City, Taiwan; b Department of Research, Taipei Tzu Chi Hospital, Buddhist Tzu Chi Medical Foundation, No. 289, Jianguo Rd, Fuxing Village, Xindian District, New Taipei City, Taiwan; c School of Biomedical Sciences, Queensland University of Technology, 2 George St, Brisbane City, Queensland, Australia

**Keywords:** TriNetX, Sleeve gastrectomy, Bariatric surgery

**Figure:**
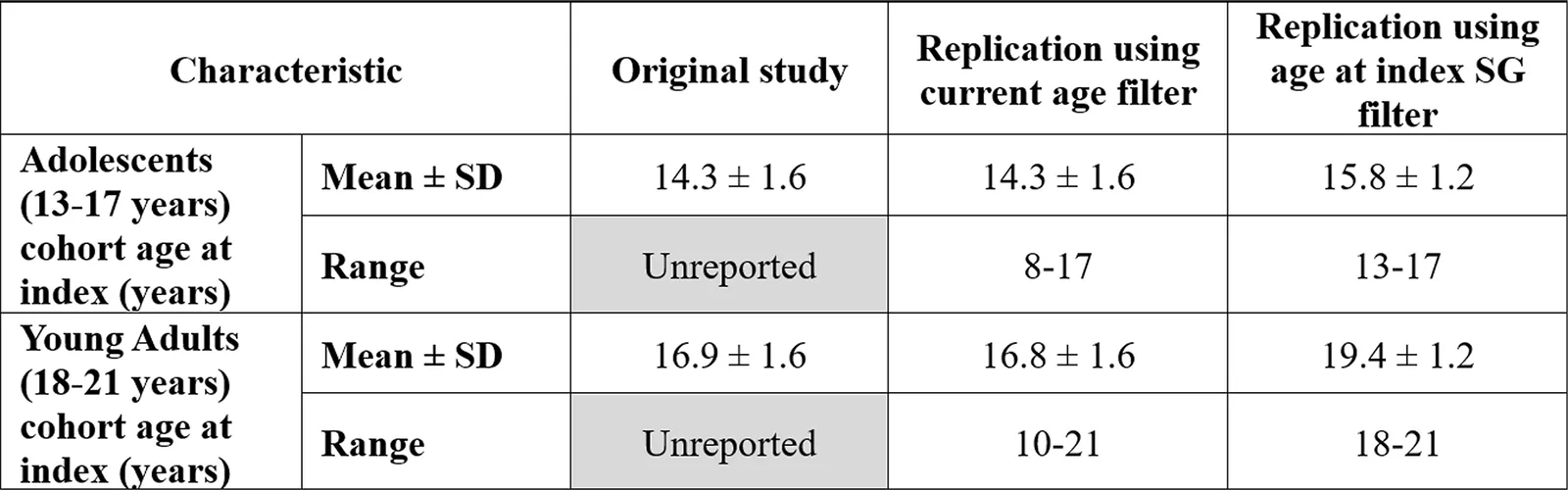

Dear editor,

We read Taki-Eldin and colleagues’ [1] retrospective study comparing laparoscopic sleeve gastrectomy results between adolescents (13–17 years) and young adults (18–21 years). We commend the authors for using a large clinical data network (TriNetX) to examine granular but increasingly common populations in obesity medicine. However, filtering cohorts by age in TriNetX can be prone to methodological error which can compromise study results [2], [3]. While the authors state that their cohorts “were defined by patient age at the index event”, their baseline characteristics appear contradictory, with the young adult (18–21 years) cohort having a mean age at index of 16.9 ± 1.6 years.

This discrepancy prompted us to attempt replications of Taki-Eldin and colleagues’ cohorts (Supplementary Tables 1–4). We performed these replications using the same network (TriNeX US Collaborative Network) and cohort definitions reported by the authors on September 10, 2026. At the time of replication, the network had approximately 135 million patients recorded, slightly larger than the 113 million patients reported in the original study.

A common error in TriNetX-based paediatric studies is to filter patients based on their current age. For example, if an adolescent patient underwent sleeve gastrectomy at 12 years of age in 2019, that patient would be 19 years old in 2026 and therefore erroneously included in the young adult cohort if a current age filter was used. When we replicated Taki-Eldin and colleagues' study using a current age filter, both cohorts returned a similar number of patients to the published study (Table 1) with nearly identical mean ages at index sleeve gastrectomy (Table 2). In contrast, when we replicated the study defining cohorts by patient age at the index event, both cohort sizes (Table 1) and age at sleeve gastrectomy (Table 2) were very different to the original study's published results. These replications therefore suggest that Taki-Eldin and colleagues may have used an incorrect age filtering approach to define their cohorts.

**Table 1:** Cohort sizes for adolescent (13–17 years) and young adult (18–21) cohorts in the published study, our replication filtering patients based on their current age, and our replication filtering patients based on their age at sleeve gastrectomy.

Cohort	Adolescents (13–17 years)	Young Adults (18–21 years)
**Original study**	327	1056
**Replication using current age filter**	351	1225
**Replication using age at index SG filter**	1596	2100

**Table 2:** Age at index sleeve gastrectomy for adolescent (13–17 years) and young adult (18–21 years) in the published study, our replication filtering patients based on their current age, and our replication filtering patients based on their age at sleeve gastrectomy.

Characteristic		Original study	Replication using current age filter	Replication using age at index SG filter
**Adolescents (13–17 years) cohort age at index (years)**	**Mean ± SD**	14.3 ± 1.6	14.3 ± 1.6	15.8 ± 1.2
	**Range**	Unreported	8–17	13–17
**Young Adults (18–21 years) cohort age at index (years)**	**Mean ± SD**	16.9 ± 1.6	16.8 ± 1.6	19.4 ± 1.2
	**Range**	Unreported	10–21	18–21

This error is consequential to the study. In our replicated cohorts using the incorrect ‘current age filter’, 826/1225 (68.5%) of the patients in the young adult cohort were actually below 18 years of age at the time of their sleeve gastrectomy; 40/351 (11.4%) of the patients in the adolescent cohort were actually below 13 years of age at the time of their sleeve gastrectomy. This suggests that both cohorts in Taki-Eldin and colleagues' study consist mostly of pediatric patients. This may therefore underestimate differences in post-operative outcomes between adolescent and young adult cohorts. Additionally, using a correct age filtering method retains suitable patients that would be erroneously excluded with a current age filter, meaning that the authors' hypothesis could be tested on a much larger patient sample. We would therefore encourage the authors to re-run their analysis defining their cohorts based on their age at the time of sleeve gastrectomy.

## Author contributions

Joshua Wang performed the partial replication. Joshua Wang and Kian-Hwee Chong analysed the data. Joshua Wang drafted the manuscript. Both authors reviewed the manuscript and agreed to be accountable for its contents.

## Author disclosures

Joshua Wang has no conflicts of interest to declare. Kian-Hwee Chong has no conflicts of interest to declare.

## Ethical adherance

N/A - This letter is a partial re-analysis of a published study.

## Generative AI disclosure

During the preparation of this work the authors did not use AI. Only human intelligence was used to write this letter.

## Source of funding

Beyond payment to the research staff by the 10.13039/501100008108Taipei Tzu Chi Hospital, this research did not receive any specific grant from funding agencies in the public, commercial, or not-for-profit sectors.

## Declaration of competing interest

The authors declare that they have no known competing financial interests or personal relationships that could have appeared to influence the work reported in this paper.
