# Supplementary material for: Cohort definition concerns in a TriNetX study comparing adolescent and young adult sleeve gastrectomy outcomes
Source: Obes Pillars. 2026 Oct 30;20:100349. doi: 10.1016/j.obpill.2026.100349 (PMC13753582; doi:10.1016/j.obpill.2026.100349)
Supplement: Supplementary file 1 [file mmc1.docx]

**Supplementary material**

Supplementary Table 1: Exported cohort definition for replicating the adolescent cohort using a current age filter. This query was run on the TriNetX US Collaborative Network on the 10^th^ September 2026 and returned 351 eligible patients

| Ungrouped terms | | | | | |
| --- | --- | --- | --- | --- | --- |
|  | must have |  | demographics | Age | Age (between 13 and 17 years (most recent occurrence)) |
|  |  | and | procedure | UMLS:CPT:43775 | Laparoscopy, surgical, gastric restrictive procedure; longitudinal gastrectomy (ie, sleeve gastrectomy) |
|  |  | and | diagnosis | UMLS:ICD10CM:E66.01 | Morbid (severe) obesity due to excess calories |
|  | cannot have |  | procedure | UMLS:CPT:43644 | Laparoscopy, surgical, gastric restrictive procedure; with gastric bypass and Roux-en-Y gastroenterostomy (roux limb 150 cm or less) |
|  |  | or | procedure | UMLS:CPT:43645 | Laparoscopy, surgical, gastric restrictive procedure; with gastric bypass and small intestine reconstruction to limit absorption |
|  |  | or | procedure | UMLS:CPT:43846 | Gastric restrictive procedure, with gastric bypass for morbid obesity; with short limb (150 cm or less) Roux-en-Y gastroenterostomy |
|  |  | or | procedure | UMLS:CPT:43847 | Gastric restrictive procedure, with gastric bypass for morbid obesity; with small intestine reconstruction to limit absorption |
|  |  | or | procedure | UMLS:CPT:43770 | Laparoscopy, surgical, gastric restrictive procedure; placement of adjustable gastric restrictive device (eg, gastric band and subcutaneous port components) |
|  |  | or | procedure | UMLS:CPT:43845 | Gastric restrictive procedure with partial gastrectomy, pylorus-preserving duodenoileostomy and ileoileostomy (50 to 100 cm common channel) to limit absorption (biliopancreatic diversion with duodenal switch) |
|  |  | or | procedure | UMLS:CPT:43842 | Gastric restrictive procedure, without gastric bypass, for morbid obesity; vertical-banded gastroplasty |
|  |  | or | procedure | UMLS:CPT:43848 | Revision, open, of gastric restrictive procedure for morbid obesity, other than adjustable gastric restrictive device (separate procedure) |

Supplementary Table 2: Exported cohort definition for replicating the adolescent cohort using an age at index (sleeve gastrectomy) filter. This query was run on the TriNetX US Collaborative Network on the 10^th^ September 2026 and returned 1596 eligible patients

| Ungrouped terms | | | | | |
| --- | --- | --- | --- | --- | --- |
|  | must have |  | procedure | UMLS:CPT:43775 | Laparoscopy, surgical, gastric restrictive procedure; longitudinal gastrectomy (ie, sleeve gastrectomy) (between 13 and 17 years old at event) |
|  |  | and | diagnosis | UMLS:ICD10CM:E66.01 | Morbid (severe) obesity due to excess calories |
|  | cannot have |  | procedure | UMLS:CPT:43644 | Laparoscopy, surgical, gastric restrictive procedure; with gastric bypass and Roux-en-Y gastroenterostomy (roux limb 150 cm or less) |
|  |  | or | procedure | UMLS:CPT:43645 | Laparoscopy, surgical, gastric restrictive procedure; with gastric bypass and small intestine reconstruction to limit absorption |
|  |  | or | procedure | UMLS:CPT:43846 | Gastric restrictive procedure, with gastric bypass for morbid obesity; with short limb (150 cm or less) Roux-en-Y gastroenterostomy |
|  |  | or | procedure | UMLS:CPT:43847 | Gastric restrictive procedure, with gastric bypass for morbid obesity; with small intestine reconstruction to limit absorption |
|  |  | or | procedure | UMLS:CPT:43770 | Laparoscopy, surgical, gastric restrictive procedure; placement of adjustable gastric restrictive device (eg, gastric band and subcutaneous port components) |
|  |  | or | procedure | UMLS:CPT:43845 | Gastric restrictive procedure with partial gastrectomy, pylorus-preserving duodenoileostomy and ileoileostomy (50 to 100 cm common channel) to limit absorption (biliopancreatic diversion with duodenal switch) |
|  |  | or | procedure | UMLS:CPT:43842 | Gastric restrictive procedure, without gastric bypass, for morbid obesity; vertical-banded gastroplasty |
|  |  | or | procedure | UMLS:CPT:43848 | Revision, open, of gastric restrictive procedure for morbid obesity, other than adjustable gastric restrictive device (separate procedure) |

Supplementary Table 3: Exported cohort definition for replicating the young adult cohort using a current age filter. This query was run on the TriNetX US Collaborative Network on the 10^th^ September 2026 and returned 1225 eligible patients

| Ungrouped terms | | | | | |
| --- | --- | --- | --- | --- | --- |
|  | must have |  | demographics | Age | Age (between 18 and 21 years (most recent occurrence)) |
|  |  | and | procedure | UMLS:CPT:43775 | Laparoscopy, surgical, gastric restrictive procedure; longitudinal gastrectomy (ie, sleeve gastrectomy) |
|  |  | and | diagnosis | UMLS:ICD10CM:E66.01 | Morbid (severe) obesity due to excess calories |
|  | cannot have |  | procedure | UMLS:CPT:43644 | Laparoscopy, surgical, gastric restrictive procedure; with gastric bypass and Roux-en-Y gastroenterostomy (roux limb 150 cm or less) |
|  |  | or | procedure | UMLS:CPT:43645 | Laparoscopy, surgical, gastric restrictive procedure; with gastric bypass and small intestine reconstruction to limit absorption |
|  |  | or | procedure | UMLS:CPT:43846 | Gastric restrictive procedure, with gastric bypass for morbid obesity; with short limb (150 cm or less) Roux-en-Y gastroenterostomy |
|  |  | or | procedure | UMLS:CPT:43847 | Gastric restrictive procedure, with gastric bypass for morbid obesity; with small intestine reconstruction to limit absorption |
|  |  | or | procedure | UMLS:CPT:43770 | Laparoscopy, surgical, gastric restrictive procedure; placement of adjustable gastric restrictive device (eg, gastric band and subcutaneous port components) |
|  |  | or | procedure | UMLS:CPT:43845 | Gastric restrictive procedure with partial gastrectomy, pylorus-preserving duodenoileostomy and ileoileostomy (50 to 100 cm common channel) to limit absorption (biliopancreatic diversion with duodenal switch) |
|  |  | or | procedure | UMLS:CPT:43842 | Gastric restrictive procedure, without gastric bypass, for morbid obesity; vertical-banded gastroplasty |
|  |  | or | procedure | UMLS:CPT:43848 | Revision, open, of gastric restrictive procedure for morbid obesity, other than adjustable gastric restrictive device (separate procedure) |

Supplementary Table 4: Exported cohort definition for replicating the young adult cohort using an age at index (sleeve gastrectomy) filter. This query was run on the TriNetX US Collaborative Network on the 10^th^ September 2026 and returned 2100 eligible patients

| Ungrouped terms | | | | | |
| --- | --- | --- | --- | --- | --- |
|  | must have |  | procedure | UMLS:CPT:43775 | Laparoscopy, surgical, gastric restrictive procedure; longitudinal gastrectomy (ie, sleeve gastrectomy) (between 18 and 21 years old at event) |
|  |  | and | diagnosis | UMLS:ICD10CM:E66.01 | Morbid (severe) obesity due to excess calories |
|  | cannot have |  | procedure | UMLS:CPT:43644 | Laparoscopy, surgical, gastric restrictive procedure; with gastric bypass and Roux-en-Y gastroenterostomy (roux limb 150 cm or less) |
|  |  | or | procedure | UMLS:CPT:43645 | Laparoscopy, surgical, gastric restrictive procedure; with gastric bypass and small intestine reconstruction to limit absorption |
|  |  | or | procedure | UMLS:CPT:43846 | Gastric restrictive procedure, with gastric bypass for morbid obesity; with short limb (150 cm or less) Roux-en-Y gastroenterostomy |
|  |  | or | procedure | UMLS:CPT:43847 | Gastric restrictive procedure, with gastric bypass for morbid obesity; with small intestine reconstruction to limit absorption |
|  |  | or | procedure | UMLS:CPT:43770 | Laparoscopy, surgical, gastric restrictive procedure; placement of adjustable gastric restrictive device (eg, gastric band and subcutaneous port components) |
|  |  | or | procedure | UMLS:CPT:43845 | Gastric restrictive procedure with partial gastrectomy, pylorus-preserving duodenoileostomy and ileoileostomy (50 to 100 cm common channel) to limit absorption (biliopancreatic diversion with duodenal switch) |
|  |  | or | procedure | UMLS:CPT:43842 | Gastric restrictive procedure, without gastric bypass, for morbid obesity; vertical-banded gastroplasty |
|  |  | or | procedure | UMLS:CPT:43848 | Revision, open, of gastric restrictive procedure for morbid obesity, other than adjustable gastric restrictive device (separate procedure) |
